# Endothelial Autocrine Signaling through CXCL12/CXCR4/FoxM1 Axis Contributes to Severe Pulmonary Arterial Hypertension

**DOI:** 10.3390/ijms22063182

**Published:** 2021-03-20

**Authors:** Dan Yi, Bin Liu, Ting Wang, Qi Liao, Maggie M. Zhu, You-Yang Zhao, Zhiyu Dai

**Affiliations:** 1 Department of Internal Medicine, College of Medicine-Phoenix, University of Arizona, Phoenix, AZ 85004, USA; dyi@arizona.edu (D.Y.); binliu3@arizona.edu (B.L.); twang@arizona.edu (T.W.); 2 Department of Preventative Medicine, Zhejiang Provincial Key Laboratory of Pathophysiology Technology, Medical School of Ningbo University, Ningbo 315211, China; liaoqi@nbu.edu.cn; 3 Program for Lung and Vascular Biology, Stanley Manne Children’s Research Institute, Ann & Robert H. Lurie Children’s Hospital of Chicago, Chicago, IL 60611, USA; mengqizhu2021@u.northwestern.edu (M.M.Z.); youyang.zhao@northwestern.edu (Y.-Y.Z.); 4 Department of Pediatrics, Division of Critical Care, Northwestern University Feinberg School of Medicine, Chicago, IL 60611, USA; 5 Department of Pharmacology, Northwestern University Feinberg School of Medicine, Chicago, IL 60611, USA; 6 Department of Medicine, Northwestern University Feinberg School of Medicine, Chicago, IL 60611, USA

**Keywords:** hypoxia, vascular remodeling, angiogenesis, pulmonary hypertension, endothelium

## Abstract

Endothelial autocrine signaling is essential to maintain vascular homeostasis. There is limited information about the role of endothelial autocrine signaling in regulating severe pulmonary vascular remodeling during the onset of pulmonary arterial hypertension (PAH). In this study, we employed the first severe pulmonary hypertension (PH) mouse model, *Egln1^Tie2Cre^* (*Tie2Cre*-mediated disruption of *Egln1*) mice, to identify the novel autocrine signaling mediating the pulmonary vascular endothelial cell (PVEC) proliferation and the pathogenesis of PAH. PVECs isolated from *Egln1^Tie2Cre^* lung expressed upregulation of many growth factors or angiocrine factors such as CXCL12, and exhibited pro-proliferative phenotype coincident with the upregulation of proliferation-specific transcriptional factor FoxM1. Treatment of CXCL12 on PVECs increased FoxM1 expression, which was blocked by CXCL12 receptor CXCR4 antagonist AMD3100 in cultured human PVECs. The endothelial specific deletion of *Cxcl12* *(Egln1/Cxcl12^Tie2Cre^*) or AMD3100 treatment *in Egln1^Tie2Cre^* mice downregulated FoxM1 expression in vivo. We then generated and characterized a novel mouse model with endothelial specific FoxM1 deletion in *Egln1^Tie2Cre^* mice (*Egln1/Foxm1^Tie2Cre^*), and found that endothelial FoxM1 deletion reduced pulmonary vascular remodeling and right ventricular systolic pressure. Together, our study identified a novel mechanism of endothelial autocrine signaling in regulating PVEC proliferation and pulmonary vascular remodeling in PAH.

## 1. Introduction

Pulmonary arterial hypertension (PAH) is characterized by a progressive increase of vascular resistance and obstructive vascular remodeling affecting pulmonary arterioles, eventually leading to right heart failure and death [1,2]. Endothelial injury and hyperproliferation are hallmarks of PAH [3,4,5]. Healthy pulmonary vascular endothelial cells (PVECs) maintain vascular homeostasis via the preservation of vascular integrity, retaining vascular tone, and exhibiting an anti-inflammatory niche. Endothelial injury or dysfunction has been believed to be the initial event during the development of PAH [6]. Dysfunctional PVECs produce many kinds of growth factors or angiocrine factors which sustain the pro-proliferative environment. Therapies that attenuate PVEC proliferation may therefore provide benefit to PAH patients.

Both autocrine and paracrine signaling pathways are important to maintain vascular homeostasis and contribute to pathological angiogenesis [6]. PVECs from PAH patients exhibit increased production of growth factors, such as FGF2, IL-6, ET-1, TGF-beta, etc. These factors promote PVECs/smooth muscle cells (SMCs)/fibroblast proliferation and survival, stimulate SMC vasoconstriction, and even recruit leukocytes [6]. For example, our previous studies demonstrated that PVECs from PAH patients or *Egln1^Tie2Cre^* mice which develop spontaneous PH [7,8], secrete multiple angiocrine factors including CXCL12, PDGF-B, ET-1, and MIF, which induced the expression of proliferation specific transcriptional factor forkhead box M1 (FoxM1) and proliferation of SMCs. These events further lead to pulmonary vascular remodeling and PAH [9]. However, the role of the endothelial autocrine signaling and underline mechanisms in the pathogenesis of PAH remain elusive. FoxM1 is activated after tissue injury and upregulated in many solid tumors and leukemia [10]. Our previous study also showed that FoxM1 is activated for endothelial regeneration after inflammatory lung injury [11,12], which often serves as a trigger of PAH development [13]. However, the role of endothelial FoxM1 in the pathogenesis of PAH is not known.

In this study, we employed transcriptome analysis of PVECs isolated from *Egln1^Tie2Cre^* mice. Our study identified the intriguing signaling of the endothelial autocrine pathway via the CXCL12/CXCR4/FoxM1 axis, which mediates endothelial proliferation in the pathogenesis of PAH.

## 2. Results

**Figure 1:**
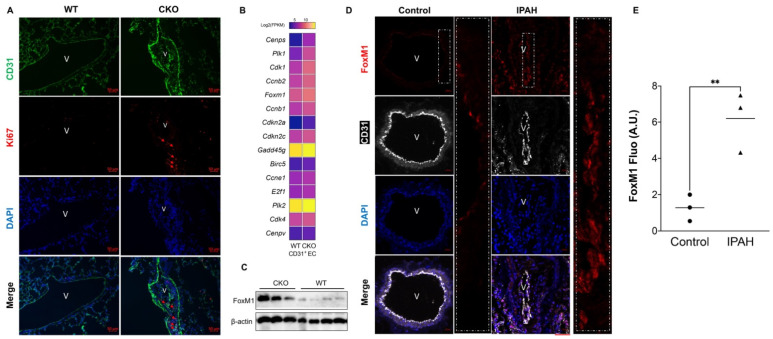
Endothelial proliferation is coincident with the upregulation of FoxM1 expression in PAH. (**A**) Immunostaining demonstrated that PVECs is pro-proliferative in *Egln1^Tie2Cre^* mice (CKO). *Egln1^f/f^* (WT) and CKO lung sections were stained with anti-Ki67 (red), a marker for cell proliferation, and anti-CD31 (green, indicative of ECs) antibodies. Red arrows indicate Ki67 positive ECs. (**B**) A heatmap of RNA sequencing analysis showed the upregulation of many genes related to cell proliferation including FoxM1 in the PVECs from *Egln1^Tie2Cre^* mice compared with PVECs from WT mice. (**C**) Western blotting showed that FoxM1 is highly induced in the lung of *Egln1^Tie2Cre^* mice. (CKO, *n* = 3; WT, *n* = 4) (**D**) Immunostaining against FoxM1 demonstrated that FoxM1 is highly expressed and induced in the lung of IPAH patients compared with failed donors (Control). (**E**) Quantification of FoxM1 expression. Immunofluorescent intensity (Fluo) was graded from 1 to 10m, with 10 being the highest. FoxM1 expression was upregulated in remodeling vessels of patients with IPAH. (Control, *n* = 3; IPAH, *n* = 3). Student *t*-test, ** *p* <0.01. A.U. indicates arbitrary units. V = vessel. Scale bar = 50 μm (**A**,**D**).

Endothelial cells (ECs) hyperproliferation is a hallmark of PAH. Our previous studies demonstrated that *Egln1^Tie2Cre^* mice exhibit severe PH, including obliterative pulmonary vascular remodeling, plexiform-like lesions, severe right heart hypertrophy, and failure [7]. To further understand the role of endothelial hyperproliferation in the pathogenesis of PAH, we confirmed that pulmonary vascular endothelial cells (PVECs) were pro-proliferative as evidenced by increased Ki67^+^/CD31^+^ cells in the pulmonary vascular bed in *Egln1^Tie2Cre^* mice (Figure 1A). We also performed whole transcriptome RNA sequencing on isolated lung PVECs (CD31^+^) from WT and *Egln1^Tie2Cre^* mice. RNA-seq analysis observed the upregulation of many genes related to cell proliferation including *Foxm1*, *E2f1*, *Cenps*, *Plk1*, *Cdk1*, *Ccnb2*, *Ccnb1*, etc., (Figure 1B), and Western blot demonstrated that FoxM1 protein expression was markedly upregulated in the lungs of *Egln1^Tie2Cre^* mice (Figure 1C). These data further support the finding that *Egln1^Tie2Cre^* lung PVECs are pro-proliferative. Previous studies from multiple groups have demonstrated that FoxM1, a key transcriptional factor for cell cycle progression, is upregulated in PASMCs and contributes to the hyperproliferation of PASMCs and vascular remodeling of PAH [9,14,15,16]. As pulmonary ECs from PAH patients share similar pro-proliferative features with PAMSCs, therefore we further examined FoxM1 expression in the lung sections from IPAH patients via immunohistochemistry staining. We observed that FoxM1 was markedly increased in pulmonary ECs in IPAH patients compared to that from control (failed donors) (Figure 1D,E and Supplemental Figure S1), suggesting that endothelial FoxM1 might be involved in the pathogenesis of PAH.

**Figure 2:**
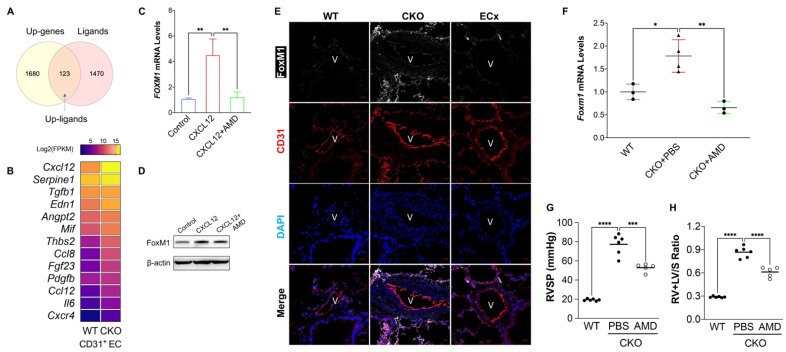
Upregulation of angiocrine factors in the PVECs from *Egln1^Tie2Cre^* mice. (**A**) A diagram showing that 123 of 1803 upregulated genes are ligands. (**B**) An RNA-seq analysis heatmap showing the representative angiocrine factors in PVECs from *Egln1^Tie2Cre^* mice. (**C**,**D**) CXCL12 treatment induced FOXM1 expression in a CXCR4 dependent manner. HLMVECs was treated with CXCL12 (100 ng/mL) and/or CXCR4 inhibitor AMD3100 (AMD, 2 μg/mL) for 8 h (**C**) and 12 h (**D**). (**E**) Immunostaining of anti-FoxM1 showed that endothelial FoxM1 was downregulated in the PVECs from *Egln1/Cxcl12^Tie2Cre^* mice (ECx), a mouse model with Cxcl12 deletion in ECs of *Egln1^Tie2Cre^* mice (CKO). (**F**) AMD3100 treatment significantly attenuated *Foxm1* expression in *Egln1^Tie2Cre^* mice. (WT, *n* = 3; CKO+PBS, *n* = 4; CKO + AMD3100, *n* = 3). (**G**,**H**) AMD3100 treatment reduced PH in *Egln1^Tie2Cre^* mice. RVSP (**G**) was measured and RV hypertrophy (**H**) was determined. (WT, *n* = 6; CKO+PBS, *n* = 6; CKO+AMD3100, *n* = 5). One-way ANOVA with Tukey post hoc analysis for multiple group comparisons. * *p* < 0.05, ** *p* < 0.01, *** *p* < 0.001, **** *p* < 0.0001. Scale bar = 20 μm (**E**).

The vascular system maintains its homeostasis by producing various kinds of factors to act on vasculature and peripheral cells. The RNA-sequencing analysis also suggests that ECs from *Egln1^Tie2Cre^* mice expressed many genes related to secretive proteins, 123 of 1680 upregulated genes are ligands according to the Secretome database [17] (Figure 2A). Many of these genes are angiocrine factors including *Cxcl12, Tgfb1, Edn1, Mif*, and *Pdgfb* (Figure 2B). Cxcl12 is the top list gene (15.6 fold: *Egln1^Tie2Cre^* vs. WT) upregulated in the PVECs from *Egln1^Tie2Cre^* mice and mediated the development of PH demonstrated by multiple studies [5,7,9,18,19,20]. Cxcl12 receptor Cxcr4 is also increased (3.2 fold: *Egln1^Tie2Cre^* vs. WT) in PVECs from *Egln1^Tie2Cre^* mice compared to *Egln1^f/f^* mice (Figure 2B). Thus, we hypothesized that endothelial autocrine signaling through CXCL12/CXCR4 mediates endothelial proliferation and the pathogenesis of PAH. To demonstrate this hypothesis, we incubated healthy human lung microvascular endothelial cells (hLMVECs) with CXCL12 and found that CXCL12 treatment significantly increased FoxM1 expression in hLMVECs, which was blocked by its receptor CXCR4 inhibitor AMD3100 (Figure 2C,D). To further determine whether CXCL12/CXCR4 signaling regulates FoxM1 expression in vivo, we examined the expression of endothelial FoxM1 in *Egln1/Cxcl12^Tie2Cre^* mice, an endothelial-specific deletion of Cxcl12 in the *Egln1^Tie2Cre^* mice [7,9]. We found that FoxM1 in the PVECs was reduced in *Egln1/Cxcl12^Tie2Cre^* mice compared to *Egln1^Tie2Cre^* mice, accessed by immunofluorescent staining against FoxM1 (Figure 2E). We also observed that FoxM1 expression was attenuated by blocking CXCR4 signaling via AMD3100 treatment in *Egln1^Tie2Cre^* mice (Figure 2F), which was consistent with reduction of RVSP and RV/(LV + S) ratio in *Egln1^Tie2Cre^* mice by AMD3100 treatment (Figure 2G,H). Taken together, our studies suggest that endothelial autocrine CXCL12/CXCR4 signaling pathway regulates FoxM1 expression in PVECs during PAH.

**Figure 3:**
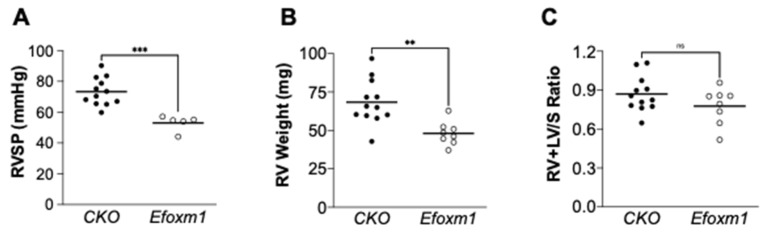
Endothelial *Foxm1* deletion protected from *Egln1* deficiency-induced PH. (**A**) RV hemodynamic measurement showed that endothelial *Foxm1* deletion (*Egln1/Foxm1^Tie2Cre^*, *Efoxm1*) attenuated RVSP in *Egln1^Tie2Cre^* mice (CKO). (CKO, *n* = 12; Efoxm1, *n* = 5). RVSP data from 3 *Efloxm1* mice were not added due to failed RVSP measurement. (**B**) Cardiac dissection demonstrated that *Foxm1* disruption in *Egln1^Tie2Cre^* mice reduced RV weight compared with *Egln1^Tie2Cre^* mice. (CKO, *n* = 12; Efoxm1, *n* = 8). (**C**) The RV/(LV+S) ratio was not changed in *Egln1/Foxm1^Tie2Cre^* compared with *Egln1^Tie2Cre^* mice. (CKO, *n* = 12; Efoxm1, *n* = 8). Student *t*-test, ** *p* < 0.01, *** *p* < 0.001, ns: not significant.

Our previous study demonstrated that FoxM1 is required for endothelial regeneration and repair after inflammatory induced acute lung injury and responded to CXCL12 treatment [21]. The role of endothelial FoxM1 in the development of PAH remains elusive. To determine whether endothelial FoxM1 is involved in the development of severe PH in *Egln1^Tie2Cre^* mice, we generated a novel mouse model with *Foxm1* deletion in ECs in *Egln1^Tie2Cre^* mice (*Egln1/Foxm1^Tie2Cre^*) via breeding *Foxm1* floxed mice with *Egln1^Tie2Cre^* mice [9,11,21]. RV hemodynamic measurement showed that *Egln1/Foxm1^Tie2Cre^* mice exhibited a reduction of the right ventricular systolic pressure (RVSP), an indicator of pulmonary arterial pressure, compared to age and gender-matched *Egln1^Tie2Cre^* mice (Figure 3A), suggesting that PH was attenuated in *Egln1/Foxm1^Tie2Cre^* mice. Cardiac dissection showed that RV weight was significantly reduced in *Egln1/Foxm1^Tie2Cre^* mice compared to *Egln1^Tie2Cre^* mice (Figure 3B). However, we did not observe a significant change of the weight ratio of RV vs. left ventricle plus septum (RV/LV+S) between *Egln1/Foxm1^Tie2Cre^* and *Egln1^Tie2Cre^* mice (Figure 3C).

**Figure 4:**
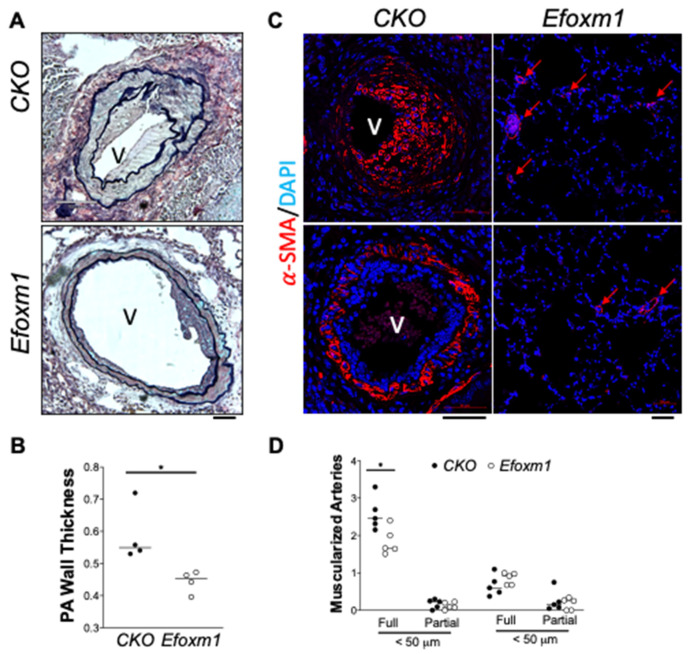
*Foxm1* deletion in ECs attenuated pulmonary vascular remodeling in *Egln1^Tie2Cre^* mice. (**A**,**B**) Representative images of Russell−Movat pentachrome staining showing the reduction of pulmonary wall thickness in *Egln1/Foxm1^Tie2Cre^* mice (*Efoxm1*). (CKO, *n* = 4; Efoxm1, *n* = 4). (**C**,**D**) Immunostaining against α-SMA demonstrated attenuation of pulmonary vascular remodeling including neointima and muscularization of distal PAs in *Egln1/Foxm1^Tie2Cre^* mice. Red arrows indicate the muscularization of distal PAs. (CKO, *n* = 5; Efoxm1, *n* = 5). V = vessel. Student *t*-test, * *p* < 0.05. Scale bar = 50 μm (**A**,**C**).

We further examined the pulmonary histology via Russell−Movat pentachrome staining and anti-α-SMA staining. We found that *Egln1/Foxm1^Tie2Cre^* mice had reduced pulmonary artery (PA) wall thickness when compared to *Egln1^Tie2Cre^* mice (Figure 4A,B). Muscularization of distal PAs assessed by anti-α-SMA staining was markedly reduced in *Egln1/Foxm1^Tie2Cre^* mice compared with *Egln1^Tie2Cre^* mice (Figure 4C,D). Taken together, our data demonstrate that endothelial FoxM1 contributes to pulmonary vascular remodeling and PH in mice.

## 3. Discussion

The present study has demonstrated the autocrine signaling pathway from PVECs through CXCL12/CXCR4 signaling mediates the transcriptional factor FoxM1 expression and endothelial hyperproliferation in PAH. We found that FoxM1 is upregulated in the lung ECs of IPAH patients and contributes to the hemodynamic increase and pulmonary vascular remodeling of PAH. Understanding the role of endothelial autocrine signaling and endothelial FoxM1 in PAH pathogenesis will pave the way for FoxM1-targeted new therapies in PAH patients.

Vascular modeling in PAH involves many cell types including EC, SMCs, fibroblasts, and macrophages. Similar to the tumor microenvironment, PVECs in the lung produce many pro-proliferative factors, which sustain vascular cells (including ECs) proliferation. Our transcriptome data demonstrated that upregulation of many growth factors (*Cxcl12*, *Edn1*, *Mif*, *Pdgfb*), transcription factors (*Foxm1*, *E2f1*), and cell cycle-related genes (*Cenps*, *Plk1*, *Cdk1*, *Ccnb2*, *Cdkn2a*) in the isolated PVECs from *Egln1^Tie2Cre^* mice compared to WT mice, suggest that the autocrine signaling pathways are mediating PVEC proliferation in *Egln1^Tie2Cre^* mice.

CXCL12, also named stromal cell-derived factor 1 (SDF1), is an angiogenic chemokine that acts via its cognate receptor CXCR4 or CXCR7. Multiple studies have demonstrated that CXCL12 promoted neovascularization and angiogenesis in several organs including skeletal muscle and cardiac arterial development [22,23]. In pathological conditions, CXCL12 was shown to promote tumor and leukemia progression [24,25] and accelerate atherosclerosis [26]. Previous studies also showed that plasma CXCL12 was associated with an unfavorable prognosis in PAH patients [27]. Multiple studies including ours demonstrated that CXCL12/CXCR4 and CXCL12/CXCR7 pathways were involved in the development of hypoxia and *Egln1* deficiency-induced PH [7,9,20,28], and blocking CXCL12 signaling attenuated PH in mice and rats [19,28,29]. In the present study, we found that Cxcl12 was highly expressed in PVECs and markedly upregulated in the PVECs from *Egln1^Tie2Cre^* mice. Treatment of CXCL12 on PVECs induced FoxM1 expression through CXCR4. Taken together, our data showed that endothelial autocrine signaling through CXCL12/CXCR4/FoxM1 mediated PVEC proliferation in PAH. Moreover, Bordenave et al. showed that CXCR7 was overexpressed in the pericytes of experimental PH and human PAH. Their studies demonstrated that the binding of CXCL12 to CXCR7 promoted pericyte recruitment of blood vessel remodeling. [20] This mechanism might partly explain the pulmonary vascular remodeling observed in the *Egln1^Tie2cre^* mice.

Proliferation specific transcription factor FoxM1 has been shown to regulate EC, SMC, and fibroblast proliferation in the disease model of acute lung injury [11,21], PH [9,14,15,16] and interstitial lung fibrosis [11], respectively. A local PAH microenvironment is needed to maintain FoxM1 expression. In our previous studies, we first checked the expression of FoxM1 in cultured PAEC from IPAH patients and failed donors, but we did not observe a significant change of FoxM1 protein expression [9]. This might be because cultured cells might lose the endogenous feature in vivo [30]. In the present studies, we observed that endothelial FoxM1 is upregulated in the PVECs of IPAH patients and severe PH mouse model *Egln1^Tie2Cre^* mice, a mouse model with marked elevation of RVSP, severe RV hypertrophy, and obliterative vascular remodeling, resembling many pathological features of IPAH in patients. Genetic disruption of endothelial FoxM1 in *Egln1^Tie2Cre^* mice reduced RVSP, and attenuated pulmonary vascular remodeling evident by reduction of PA wall thickness and muscularization of distal PAs, suggesting FoxM1 in ECs contributes to the severity of vascular remodeling and PAH. This is different from our previous observation that *Foxm1^Tie2Cre^* mice did not show protection from hypoxia (10% O_2_, 3 weeks)-induced PH [9]. This might be due to the fact that hypoxia does not stimulate significant EC proliferation in mice and induces mild pulmonary vascular remodeling and PH. Another observation in this study is that RV weight but not RV/LV + S ratio was reduced in *Egln1/Foxm1^Tie2Cre mice^* compared with *Egln1^Tie2Cre^* mice, which is because LV weight is also reduced in *Egln1/Foxm1^Tie2Cre^* mice compared with *Egln1^Tie2Cre^* mice. The data was not shown due to another manuscript on the topic of left ventricular hypertrophy observed in *Egln1^Tie2Cre^* mice. Another limitation of this study is that neither systemic blood pressure nor cardiac output were assessed in these studies. Further studies are warranted to determine whether endothelial FoxM1 is involved in right heart failure.

Our previous studies demonstrated that multiple growth factors (PDGF-B, CXCL12, MIF, and ET-1) were derived from dysfunctional PVECs in PH, which induced FoxM1 expression in PASMCs and activated PASMC proliferation and PH via paracrine signaling [9]. Smooth muscle cell-specific deletion of FoxM1 protected from Sugen5416/hypoxia-induced PH. Pharmacologic inhibition of FoxM1 using Thiostrepton inhibited severe PH in both Sugen 5416/hypoxia and monocrotaline-challenged rats [9]. Differently, the present study investigated the endothelial autocrine effect in PH. We generated a novel mouse model with an endothelial-specific FoxM1 deletion in *Egln1^Tie2Cre^* mice (*Egln1/Foxm1^Tie2Cre^*), and found that PVECs-derived CXCL12 by acting on its receptor CXCR4 promoted FoxM1 expression in PVECs, leading to PVECs proliferation and pulmonary vascular remodeling and PH. Taken together, our studies demonstrated that FoxM1 is a novel therapeutic target for the treatment of PAH patients.

Endothelial FoxM1 has been shown to regulate embryonic development and endothelial proliferation and repair following LPS-induced vascular injury [11,31]. Recent studies also suggest that FoxM1 is a critical driver of TGF-β-induced endothelial-to-mesenchymal transition (EndoMT) [32]. Tang et al. demonstrated that *Egln1* deficiency in ECs induced EndoMT in a HIF-2α dependent manner in vivo and in vitro [33], it is intriguing to determine whether endothelial FoxM1 is also involved in EndoMT in *Egln1^Tie2Cre^* mice contributing to severe PH. We also found that CXCL12 induced FoxM1 expression in lung ECs via p110γ PI3K→ FoxO1 signaling and mediating endothelial regeneration in sepsis-induced inflammatory lung injury [21]. BRD4 and FoxO1 have been shown to positively and negatively regulate FoxM1, respectively [34], however, our data showed that both BRD4 and FoxO1 were downregulated in the lungs of *Egln1^Tie2Cre^* mice (data not shown), suggesting that FoxO1 signaling but not BRD4 might be the direct regulator of FoxM1 in ECs.

In summary, the present study demonstrates that endothelial autocrine signaling through CXCL12/CXCR4 mediates FoxM1 induction in PVECs and contributes to endothelial proliferation and severe pulmonary vascular remodeling in PAH. This study further suggests that FoxM1 inhibition could be a therapeutic approach for PAH patients.

## 4. Materials and Methods

### 4.1. Human Samples

The human lung tissues from IPAH patients and control subjects (failed donors) were provided by the Pulmonary Hypertension Breakthrough Initiative (PHBI) and used as described previously [9,35,36]. Control lung samples were obtained from donors not found to have an appropriate recipient [36]. A table summarizing clinical and demographic characteristics of PAH patients and control are provided in Supplemental Table S1. The human lung microvascular ECs (hLMVECs) were obtained from Lonza (Alpharetta, GA, USA). The use of human samples was approved by the University of Arizona Institutional Review Board (IRB# 1907824872, approved date 26 July 2019).

### 4.2. Mice

*Egln1^Tie2Cre^*, *Egln1/Cxcl12^Tie2Cre^* mice were generated as described previously [7]. *Foxm1* floxed mice [11] were bred with *Egln1^Tie2Cre^* mice to generate *Egln1/Foxm1^Tie2Cre^* mice. For CXCR4 inhibitor AMD3100 treatment study, *Egln1^Tie2Cre^* mice at the age of 3 weeks were treated with vehicle (PBS) or CXCR4 inhibitor AMD3100 (7.5 mg/kg, daily) (MilliporeSigma, St. Louis, MO, USA) for 5 weeks. Right ventricular systolic pressure (RVSP) in mice was measured as described previously [7,9,35]. The experiments were conducted according to the National Institutes of Health guidelines on the use of laboratory animals. The animal care and study protocol were approved by the Institutional Animal Care and Use Committee of Northwestern University (#IS00006960, approved date 15 September 2017) and the University of Arizona (#19-513, approved date 15 August 2019).

### 4.3. Endothelial Cells Isolation and RNA Sequencing Analysis

Mouse lung ECs were isolated as described previously [9,35]. Purified endothelial cells (EC, CD31^+^ cells) were lysed for RNA isolation with the RNeasy mini kit (Qiagen Inc., Germantown, MD, USA) including DNase I digestion. Equal amounts of RNA from ECs isolated from three individual WT or *Egln1^Tie2Cre^* mice were pooled and sequenced with NovaSeq PE150 at Novogene Corporation Inc. (Sacramento, CA, USA) The original sequencing data were trimmed using FASTX and aligned to the reference genome using TopHat2. The differential expression analysis was performed using Cuffdiff software [37].

### 4.4. Immunofluorescent Staining and Histological Assessment

For immunofluorescent staining of cryosections, human IPAH patients and failed donors were fixed with 4% paraformaldehyde for 20 min. The sections were blocked with 5% goat serum and incubated with anti-FoxM1 (Cat # sc-376471, 1:25, Santa Cruz Biotechnology, Santa Cruz, CA, USA) and anti-CD31 (Cat#ab28364, 1:40, Abcam, Cambridge, UK) were incubated at 4 °C overnight, then incubated with Alexa 594 conjugated anti-mouse and Alexa 647 conjugated anti-rabbit IgG (Thermo Fisher Scientific, Waltham, MA, USA) at room temperature for 1 h. Nuclei were counterstained with DAPI (SouthernBiotech, Birmingham, AL, USA).

For immunofluorescent staining of cryosections from mouse lung tissue, mouse lung tissue was perfused with PBS and inflated with 50% OCT in PBS for sectioning. Staining was performed as described above.

For immunofluorescent staining of paraffin sections from mouse lung tissues, mouse lung tissues were perfused with PBS, followed by fixation and inflation with 10% formalin via tracheal instillation for routine tissue processing. The sections were dewaxed and dehydrated, followed by antigen retrieval using Antigen Unmasking Solution (# H-3300-250, Vector Lab, Burlingame, CA, USA) according to the manual. Similarly, after antigen retrieval, mouse lung sections were incubated with anti-α-SMA (Cat #ab5694, 1:300, Abcam, Cambridge, UK) at 4 °C overnight and then incubated with Alexa 594 conjugated anti-rabbit IgG (Thermo Fisher Scientific, Waltham, MA, USA) at room temperature for 1 h. For histology assessment, lung sections were dewaxed, dehydrated, and stained with a Russell−Movat pentachrome staining kit (Cat #KTRMP, StatLab, McKinney, TX, USA) according to the manufacturer’s instructions. PA wall thickness was measured using Pentachrome stained sections as described previously [9,35].

### 4.5. RNA Isolation and Quantitative Real-Time Polymerase Chain Reaction (QRT-PCR) Analysis

Total RNA was isolated from frozen mouse lung tissues with Trizol reagents (Thermo Fisher Scientific, Waltham, MA, USA) followed by cleaning-up with RNeasy Mini kit including DNase I digestion (Qiagen, Germantown, MD, USA). RNA from hLMVECs were directly isolated with RNeasy Mini kit. One microgram of RNA was transcribed into cDNA using the high-capacity cDNA reverse transcription kits (Thermo Fisher Scientific, Waltham, MA, USA) according to the manufacturer’s protocol. QRT-PCR was performed on QuantStudio 3 Real-Time PCR System (Thermo Fisher Scientific, Waltham, MA, USA) with PowerUp SYBR Green Master Mix (Thermo Fisher Scientific, Waltham, MA, USA). Target mRNA was determined using the comparative cycle threshold method of relative quantitation. Cyclophilin was used as an internal control for analysis of expression of mouse genes, while 18s rRNA gene was used for human genes. The primer sequences are provided in Supplemental Table S2.

### 4.6. Western Blot Analysis

Lung tissue samples from WT and Egln1^Tie2Cre^ mice at the age of 3.5 months were homogenized in RIPA lysis buffer supplemented with 1 mM phenylmethylsulfonyl fluoride (PMSF, MilliporeSigma, St. Louis, MO, USA) and protease inhibitor cocktails (MilliporeSigma, St. Louis, MO, USA). For in vitro cell cuture, hLMVECs were starved with serum free medium, followed by treatment with recombinant human CXCL12 (100 ng/mL, Cat #6448-SD, R&D Systems, Minneapolis, MN) or AMD3100 (2 μg/mL, MilliporeSigma, St. Louis, MO, USA). Western blot analysis was performed using anti-FoxM1 (Cat #sc-376471, 1:250, Santa Cruz Biotechnology, Santa Cruz, CA, USA). Anti-β-actin (1:10,000, Cat #A2228, MilliporeSigma, St. Louis, MO, USA) was used as a loading control.

### 4.7. Statistical Analysis

Statistical significance was determined by one-way ANOVA with a Tukey post hoc analysis that calculates P values corrected for more than 2 group comparisons using Prism 9 (Graphpad Software, Inc., San Diego, CA, USA). Two-group comparisons were analyzed by the unpaired two-tailed Student’s *t* test. *p* less than 0.05 denoted the presence of a statistically significant difference.

## Data Availability

The RNA sequencing data was available in Figshare (https://figshare.com/projects/Endothelial_Autocrine_Signaling_Contributes_to_Severe_Pulmonary_Arterial_Hypertension/99824).

## Supplementary Materials

The following are available online at https://www.mdpi.com/1422-0067/22/6/3182/s1, Figure S1: Representative images of immunostaining against FoxM1 in lower magnification. Immunostaining against FoxM1 demonstrated that FoxM1 is highly expressed and induced in the lung of IPAH patients compared with failed donors (Control). Scale bar = 50 μm, Table S1: Clinical and demographic characteristics of PAH patients and control, Table S2: Primer sequences for QRT-PCR analysis.
