# Supplementary material for: Endothelial Autocrine Signaling through CXCL12/CXCR4/FoxM1 Axis Contributes to Severe Pulmonary Arterial Hypertension
Source: Int J Mol Sci. 2021 Mar 20;22(6):3182. doi: 10.3390/ijms22063182 (PMC8003962; doi:10.3390/ijms22063182)
Supplement: Supplementary file 1 [file ijms-22-03182-s001.pdf]

SUPPLEMENTAL MATERIALS

Endothelial Autocrine Signaling through CXCL12/CXCR4/FoxM1 Axis Contributes to Severe Pulmonary Arterial Hypertension

Dan Yi, Bin Liu, Ting Wang, Qi Liao, Maggie M. Zhu, You-Yang Zhao and Zhiyu Dai

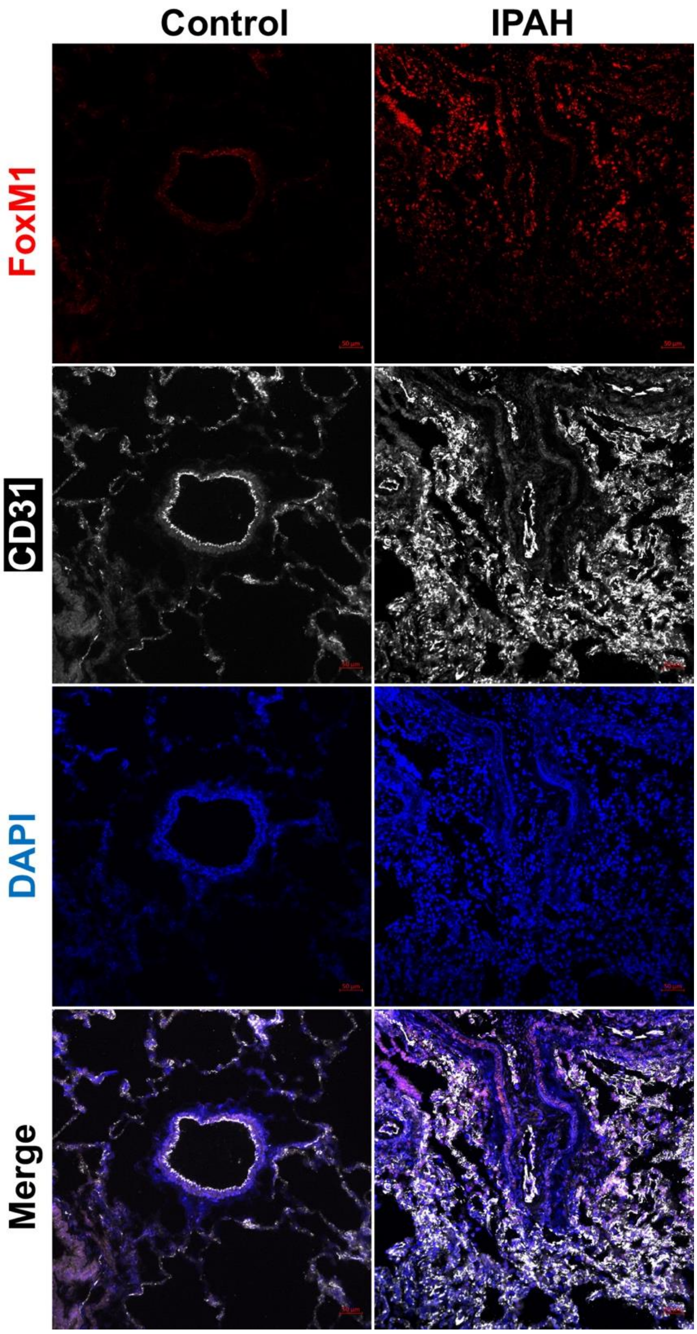

Supplemental Figure S1. Representative images of immunostaining against FoxM1 in lower magnification. Immunostaining against FoxM1 demonstrated that FoxM1 is highly expressed and induced in the lung of IPAH patients compared with failed donors (Control). Scale bar = 50 μm.

Supplemental Table S1. Clinical and demographic characteristics of PAH patients and control.

ID	PAH or Failed Donor	Gender	Race	Ethnicity	Age	WHO Functional Class	6MWD (m)	mPAP (mmHg)	CO (mL/min)	PVR (WU)	Treatment
PHBI-ST-036	IPAH	Female	White	Non-Hispanic	11	No data	243.8	95	No data	No data	Sildenafil/Ambrisentan/ Epoprostenol/Treprostinil
PHBI-ST-042	IPAH	Male	White	Non-Hispanic	53	IV	77.7	45	4.28	8.18	Tadalafil/Epoprostenol/ Sildenafil/Macitentan
PHBI-UA-026	IPAH	Female	White	Non-Hispanic	55	II	381	52	5.59	7.33	Treprostinil/Ambrisentan/ Tadalafil
PHBI-AH-022	Failed donor	Female	White	Non-Hispanic	57	N/A	N/A	N/A	N/A	N/A	N/A
PHBI-BA-044	Failed donor	Male	White	Non-Hispanic	30	N/A	N/A	N/A	N/A	N/A	N/A
PHBI-BA-059	Failed donor	Female	Asian	Non-Hispanic	34	N/A	N/A	N/A	N/A	N/A	N/A

Supplemental Table S2. Primer sequences for QRT-PCR analysis.

Gene name	Forward primer	Reverse primer
hFOXM1	GGAGGAAATGCCACACTTAGCG	TAGGACTTCTTGGGTCTTGGGGTG
h18S rRNA	TTCCGACCATAAACGATGCCGA	GACTTTGGTTTCCCGGAAGCTG
mFoxm1	CACTTGGATTGAGGACCACTT	GTCGTTTCTGCTGTGATTCC
mCyclophilin	GGCAAATGCTGGACCAAACAC	TTCCTGGACCCAAAACGCTC
